# Executive Summary: The 2025 British Society for Rheumatology guideline for the treatment of axial spondyloarthritis with biologic and targeted synthetic DMARDs

**DOI:** 10.1093/rheumatology/keaf090

**Published:** 2025-04-09

**Authors:** Sizheng Steven Zhao, Stephanie R Harrison, Ben Thompson, Max Yates, Joe Eddison, Antoni Chan, Nick Clarke, Nadia Corp, Charlotte Davis, Lambert Felix, Kalveer Flora, William J Gregory, Gareth T Jones, Christopher A Lamb, Helena Marzo-Ortega, Daniel J Murphy, Harry Petrushkin, Virinderjit Sandhu, Raj Sengupta, Stefan Siebert, Danielle A Van Der Windt, Dale Webb, Zenas Z N Yiu, Karl Gaffney

**Affiliations:** Centre for Musculoskeletal Research, Division of Musculoskeletal and Dermatological Science, School of Biological Sciences, Faculty of Biological Medicine and Health, The University of Manchester, Manchester Academic Health Science Centre, Manchester, UK; NIHR Manchester Biomedical Research Centre, Manchester University NHS Foundation Trust, Manchester, UK; Leeds Institute of Rheumatic and Musculoskeletal Medicine, University of Leeds, Leeds, UK; Leeds NIHR Biomedical Research Centre, Leeds Teaching Hospitals NHS Trust, Leeds, UK; Rheumatology Department, The Newcastle-upon-Tyne Hospitals NHS Foundation Trust, Newcastle upon Tyne, UK; Centre for Epidemiology, Norwich Medical School, University of East Anglia, Norwich, UK; Rheumatology Department, Norfolk & Norwich University Hospitals NHS Foundation Trust, Norwich, UK; Expert by Experience, Leeds, UK; University Department of Rheumatology, Royal Berkshire NHS Foundation Trust, Reading, UK; Expert by Experience, Norwich, UK; Primary Care Centre Versus Arthritis, School of Medicine, Keele University, Staffordshire, UK; Department of Rheumatology, The Leeds Teaching Hospitals NHS Trust, Leeds, UK; Primary Care Centre Versus Arthritis, School of Medicine, Keele University, Staffordshire, UK; Pharmacy Department, London North West University Healthcare NHS Trust, London, UK; Rheumatology Department, Salford Royal Hospital, Northern Care Alliance NHS Foundation Trust, Greater Manchester, UK; Faculty of Health and Education, Manchester Metropolitan University, Manchester, UK; Aberdeen Centre for Arthritis and Musculoskeletal Health (Epidemiology Group), University of Aberdeen, Aberdeen, UK; Translational & Clinical Research Institute, Faculty of Medical Sciences, Newcastle University, Newcastle upon Tyne, UK; Department of Gastroenterology, The Newcastle upon Tyne Hospitals NHS Foundation Trust, Newcastle upon Tyne, UK; Leeds Institute of Rheumatic and Musculoskeletal Medicine, University of Leeds, Leeds, UK; Leeds NIHR Biomedical Research Centre, Leeds Teaching Hospitals NHS Trust, Leeds, UK; Honiton Surgery, Department of Rheumatology, Royal Devon & Exeter Hospital, Exeter, UK; Uveitis and Scleritis Service, Moorfields Eye Hospital NHS Foundation Trust, London, UK; Department of Rheumatology, St George’s University Hospitals NHS Foundation Trust, London, UK; Royal National Hospital for Rheumatic Diseases, Royal United Hospitals, Bath, UK; School of Infection and Immunity, University of Glasgow, Glasgow, UK; Primary Care Centre Versus Arthritis, School of Medicine, Keele University, Staffordshire, UK; National Axial Spondyloarthritis Society (NASS), London, UK; Dermatology Centre, Salford Royal Hospital, Northern Care Alliance NHS Foundation Trust, Salford, UK; Rheumatology Department, Norfolk & Norwich University Hospitals NHS Foundation Trust, Norwich, UK

**Keywords:** axSpA, ankylosing spondylitis, biologics, targeted synthetic DMARDs, treatment, management, guideline, recommendations, extra-musculoskeletal manifestations, uveitis, psoriasis, inflammatory bowel disease

## Abstract

**Figure:**
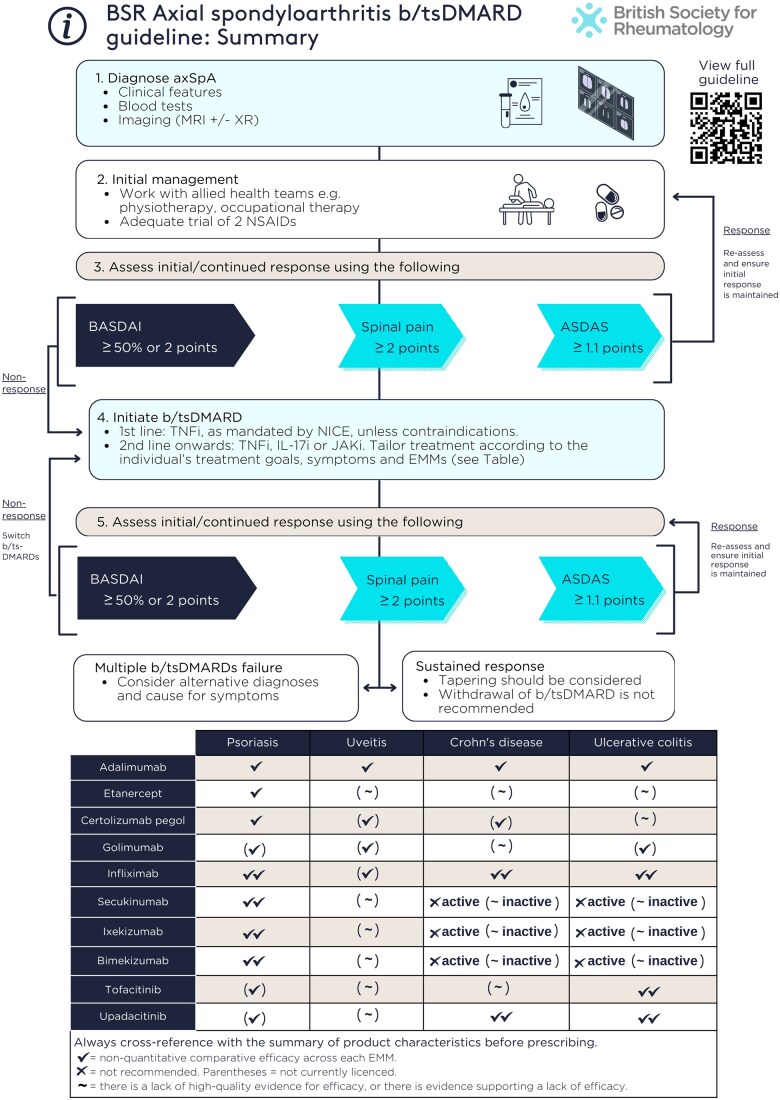


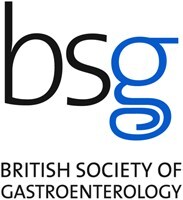



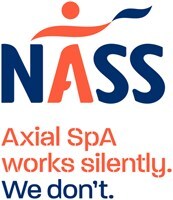



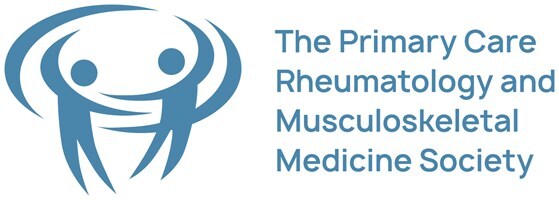



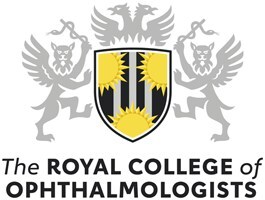



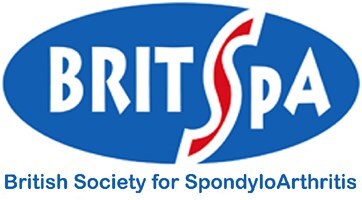


## Background and rationale for guideline development

Axial spondyloarthritis (axSpA) is a chronic inflammatory condition that predominantly affects the spine and sacroiliac joints [1]. It can also involve peripheral joints and entheses, and extra-musculoskeletal manifestations (EMMs) such as acute anterior uveitis, psoriasis and IBD. Since the previous version of the British Society for Rheumatology (BSR) axSpA guideline [2], which only included TNF inhibitors, pharmacological treatment options and strategies for axSpA have expanded, alongside rapid advancements in the treatment of EMMs as index conditions. In this increasingly complex and evolving therapeutic landscape, we aimed to update the guideline for health professionals in the UK who care for adults with axSpA.

## Guideline development

This guideline was developed in accordance with the BSR Creating Guidelines Protocol (v5.4). The guideline working group (GWG) drafted the guideline scope [3], which served as the foundation for an evidence review to support the development of recommendations. Following the GRADE process, recommendations were rated based on the strength of recommendation (1: strong, 2: conditional), the quality of evidence (A: high, B: moderate, C: low/very low) and the strength of agreement (SoA, 1–100%). Recommendations were revised until the mean and individual SoA ratings exceeded 80%.

The guideline was developed by a multidisciplinary guideline working group (GWG), comprising and reflecting the views of individuals with lived experience of axSpA, rheumatologists, an ophthalmologist, a dermatologist, a gastroenterologist, a general practitioner, an epidemiologist, a specialist nurse, a consultant physiotherapist, a specialist pharmacist and the Chief Executive Officer (CEO) of the patient-focused charity National Axial Spondyloarthritis Society (NASS). The systematic literature review was conducted by researchers with evidence synthesis expertise (L.F., N.C., D.v.d.W.).

This guideline does not cover the use of NSAIDs, glucocorticoids or conventional synthetic DMARDs; the treatment of enthesitis/spondylitis-related juvenile idiopathic arthritis; axial disease in psoriatic arthritis [4]; the safety of targeted therapies [5] or their use in pregnancy [6]; or health economic considerations.

## The guideline

This executive summary distils the recommendation statements, which must be succinct by necessity. The GWG strongly advises that these statements be interpreted alongside the supporting text published in the full guidelines and online supplementary materials (Supplementary Data S1–S6, available at *Rheumatology* online). For brevity, we refer to biologic and targeted synthetic DMARDs as “targeted therapies” throughout.

### Overarching principles

1. The primary goal of treatment for people living with axSpA is to enable them to lead healthy and productive lives by optimizing health-related quality of life through comprehensive management of all disease manifestations, prevention of structural damage, preservation of physical function, work productivity and social participation (SoA 99%).
2. Management decisions should be developed in partnership with the individual living with axSpA based on their needs and priorities, within the available resources (SoA 99%).
3. Management should involve a multidisciplinary team (MDT) coordinated by a rheumatologist, utilizing a holistic approach that incorporates both pharmacological and non-pharmacological interventions (SoA 98%).

The focus of treatment is to optimize health-related quality of life by placing the person living with axSpA at the centre of care provision. The decision to start or change targeted therapies should be overseen by the responsible consultant rheumatologist and made in partnership with the person with axSpA, taking into account individual needs and priorities. Providing information and education is essential to enable meaningful engagement in shared decision-making. Treatment goals should be reviewed regularly to ensure they remain realistic, achievable and acceptable to the person with axSpA. In the presence of EMMs, holistic management should include cross-speciality collaboration. When selecting targeted therapies, consider that people with axSpA may prioritize controlling some disease manifestations over others. Management of comorbidities should adopt an MDT approach (e.g. nurse-led annual review of cardiovascular and fracture risk, clinical psychology for mental health) in close collaboration with primary care.

Escalation to targeted therapies should not diminish the focus on non-pharmacological management. Although it is beyond the scope of this guideline to make recommendations for non-pharmacological therapies, the GWG emphasizes the importance of (1) physical activity, supervised exercise and physiotherapy, (2) aquatic physiotherapy and hydrotherapy, (3) psychological therapies and (4) supported self-management.

### Recommendations

- TNF, IL-17 or JAK inhibitors are recommended for people with active axSpA who have not responded adequately despite non-pharmacological and conventional pharmacological management (1A, SoA 97%).
- Active disease should be determined by the treating clinician in the context of verified diagnosis and inflammatory disease activity, supported by validated indices such as ASDAS, BASDAI and spinal pain (1B, SoA 97%).
  There is no evidence to support recommending one class or drug over another with respect to efficacy for musculoskeletal manifestations. The decision to escalate to targeted therapies and the choice of therapy should be made with the person with axSpA and after considering prognostic factors, comorbidities [5], and EMMs (summarized in Table 1 and discussed later). These adverse prognostic factors for radiographic structural progression should be considered as part of shared decision making when initiating targeted therapies.
  For the purpose of escalation to targeted therapies, active disease should be defined after (1) appropriate use of non-pharmacological and conventional pharmacological therapies, and (2) verifying the diagnosis and inflammatory disease activity. The diagnosis of axSpA should be verified by a consultant rheumatologist. The necessary push to reduce diagnostic delay must be cautiously balanced against the potential for misdiagnosis.
  The decision to initiate therapy should not be solely based on disease indices. Nevertheless, validated measures of disease activity should be documented at the time of treatment initiation and at each follow-up. ASDAS is the only instrument shown to correlate with radiographic progression [7, 8], and the ASDAS definition of high disease activity (≥2.1) better predicts treatment response than BASDAI ≥ 4 [9, 10]. For these reasons, the GWG recommends transitioning towards regular inclusion of ASDAS in clinical practice (Table 2).
- Response to targeted therapies should be assessed using validated indices (e.g. ASDAS, BASDAI, spinal pain) 3–4 months after initiation, and every 6–12 months if treatment is continued (1B, SoA 97%).
- The absence of response to targeted therapies should prompt reassessment of the diagnosis and the extent of inflammatory disease activity (1B, SoA 100%).
- An alternative targeted therapy is recommended for individuals with active disease who cannot tolerate, do not respond to or lose response to the initial targeted therapy (1A, SoA 99%).
  Assessments should consider compliance and whether residual symptoms are related to active inflammation. Follow-up assessment of disease activity should be holistic and supported by, but not solely reliant on, disease indices. The decision to continue therapy should be made jointly between the person with axSpA and the treating clinician.
  The nature and interval of follow-up can be adjusted based on individual circumstances but should be reviewed at each visit to ensure it remains appropriate. Patient-initiated follow-up or extended follow-up intervals may be considered if the condition is well controlled and the person with axSpA has adequate education and access to a local rheumatology advice line or equivalent to promptly re-establish contact with the clinical team if necessary. Follow-up interval should not typically exceed 24 months.
  NICE recommends a BASDAI 50% or 2-unit reduction and 2-unit reduction in spinal pain [11] which, until revised, will continue to be the cornerstone of assessment. ASDAS states and improvement criteria are superior to BASDAI in differentiating levels of, and changes in, disease activity [12]. The GWG recommends that assessments incorporate ASDAS. A reduction of ≥1.1 represents a clinically important response [13].
  When using any index, assessments should consider whether residual symptoms are related to active inflammation. Repeat imaging may help to assess inflammation and other causes of persistent symptoms. Diagnosing axSpA is challenging, and clinicians should remain open to re-evaluating the original diagnosis, particularly when there is (repeated) primary non-response to targeted therapies.
  There is currently insufficient evidence to recommend a specific sequence of targeted therapies in the case of treatment failure. In the context of verified diagnosis and inflammatory disease activity, the GWG suggests that there should not be a limit to the number of sequential therapies that any individual can have.
- In the presence of moderate-to-severe or recurrent uveitis, a monoclonal TNF inhibitor is preferred over therapies with other mechanisms of action (1A, SoA 98%).
- A history of inactive uveitis is not an absolute contraindication to therapies with other mechanisms of action (2B, SoA 97%).
- If new uveitis develops in the context of well-controlled axSpA, decisions to change treatment should be made with an ophthalmologist where possible, taking into account the severity and/or frequency of uveitis flares and response to topical steroid (1B, SoA 97%).
  The severity of uveitis can vary from infrequent mild episodes to recurrent or sight-threatening disease. For moderate–severe or recurrent uveitis, the decision to commence a targeted therapy should be jointly made between rheumatology and ophthalmology as part of an MDT. In contrast to IBD, trial data do not suggest that IL-17Ai is harmful for uveitis [14]. If uveitis develops or flares in individuals with well-controlled axSpA on etanercept or IL-17Ai, severity and/or frequency of uveitis should be considered in consultation with an ophthalmologist before automatically switching therapy.
- IL-17 and monoclonal TNF inhibitors are preferred in the presence of extensive psoriasis (e.g. >10% body surface area) or severe localized psoriasis at sites associated with high functional impairment or impact (e.g. face, scalp, palms, soles, flexures, genital or nails), ideally in conjunction with a dermatologist (1A, SoA 96%).
  In people with well-controlled axSpA but inadequately controlled psoriasis, management should be discussed with a dermatologist and may not necessarily require a change in targeted therapy. Where axSpA and psoriasis are both indications for targeted therapy, control of cutaneous psoriasis can be achieved by, in order of efficacy, IL-17 inhibitors, monoclonal TNF inhibitors or etanercept [15]. The GWG recommends using at least one objective measure for assessing and monitoring psoriasis; e.g. body surface area can be estimated using the palm method, where the individual’s palm covers ∼1% of their body surface area.
- Individuals with unexplained lower gastrointestinal symptoms should be assessed by a gastroenterologist, ideally before commencing targeted therapies (1B, SoA 97%).
- In the presence of active IBD, monoclonal TNF or JAK inhibitors are preferred; IL-17 inhibitors should not be commenced (1A, SoA 99%).
- A history of inactive IBD is not an absolute contraindication to IL-17 inhibitors or etanercept (2B, SoA 97%).
  The severity, relapse frequency and prognosis of IBD (comprising Crohn’s disease and ulcerative colitis) can vary substantially and treatment decisions should be made in collaboration with gastroenterology where possible. In well-controlled axSpA, mild IBD may be managed by gastroenterology without a change in targeted therapy. Monoclonal TNF or JAK inhibitors are preferred in people with active axSpA and IBD where advanced therapies are indicated. IL-17 inhibitors should not be commenced in people with active IBD, as it may exacerbate intestinal inflammation [16]. Given the relatively limited number of drug classes for axSpA, IL-17 inhibitors can still be considered in the context of well-controlled IBD when no other options are available, but the balance of risk and benefit in these circumstances should be carefully considered with input from gastroenterology. If an IL-17 inhibitor is used, individuals and their clinicians should regularly monitor for symptoms compatible with IBD.
- Treatment should aim to achieve predefined targets agreed upon with the individual living with axSpA, using individualized therapy adjustments that consider comorbidities and inflammatory disease activity (1B, SoA 99%).
  The TICOSPA trial of treating to target (ASDAS < 2.1) in axSpA did not achieve its primary outcome (≥30% improvement in ASAS Health Index) or the majority of secondary outcomes [17]. There is insufficient evidence to recommend treating to an index-based target, which may instead lead to cycling through a limited number of targeted therapies. Therapeutic targets should be agreed upon with the person with axSpA and should consider (1) the overall number of available therapeutic options, (2) adverse prognostic factors for disease progression and treatment response and (3) extent of inflammatory disease activity.
- Tapering of targeted therapies should be considered for individuals who have achieved sustained remission (1A, SoA 98%).
- Withdrawal of a targeted therapy in the context of sustained remission is not recommended (1A, SoA 99%).

**Table 1.** Summary of the evidence for targeted therapies across extra-musculoskeletal manifestations

Biologic or targeted synthetic DMARD^a^	Review axSpA response (weeks)	Extra-musculoskeletal manifestations			
		Psoriasis	Uveitis	Crohn's disease	Ulcerative colitis
Adalimumab	12	✓	✓	✓✓	✓
Etanercept^b^	12	✓	(∼)	(∼)	(∼)
Certolizumab pegol^c^	12	✓	(✓)	(✓)	(∼)
Golimumab^d^	12	(✓)	(✓)	(∼)	✓✓
Infliximab^e^	12	✓✓	(✓)	✓✓	✓✓
Secukinumab^f^	16	✓✓	(∼)	✗active (∼inactive)	✗active (∼inactive)
Ixekizumab^f^	16-20	✓✓	(∼)	✗active (∼inactive)	✗active (∼inactive)
Bimekizumab^g^	16	✓✓	(∼)	✗active (∼inactive)	✗active (∼inactive)
Tofacitinib^h^	16	(✓)	(∼)	(∼)	✓✓
Upadacitinib^i^	16	(✓)	(∼)	✓✓	✓✓

This table is intended as a quick summary. Always cross-reference with the summary of product characteristics before prescribing. Uveitis data pertain to prevention of acute anterior uveitis incidence or flare. ‘Active/inactive’ refer to disease activity of each extra-musculoskeletal manifestation (EEM). The number of ticks provide a non-quantitative indication of comparative efficacy across each EMM. ✗: not recommended. Parentheses: not currently licenced. ∼: There is a lack of high-quality evidence for efficacy, or there is evidence supporting a lack of efficacy—see footnote for details.

a Information applies to both bio-originator and biosimilar where relevant.

b Etanercept has lower comparative efficacy for all EMMs compared with monoclonal TNFi. The risk of uveitis and IBD onset and flare is greater in etanercept than monoclonal TNFi in observational studies. Etanercept was not superior to placebo in a small RCT of Crohn’s but is unlikely to be directly detrimental to IBD; it could be considered for axSpA, following gastroenterology review.

c Certolizumab pegol is licenced for Crohn’s in the US and Europe but not in the UK. Phase III evidence is lacking for ulcerative colitis (UC), but there are single-arm studies suggesting some effectiveness.

d Golimumab has some evidence of efficacy for psoriasis (in PsA trials) but is not licenced. Phase III evidence is lacking for Crohn’s. Golimumab dosing differs for UC (requires loading dose).

e Infliximab is not licenced for non-radiographic axSpA. Subcutaneous infliximab is licenced for IBD but not for axSpA.

f According to network meta-analysis of RCTs published after the literature search cut-off date, IL-17A inhibitors are likely inferior to monoclonal TNFi, though likely superior to placebo, for uveitis. IL-17A inhibitors are not recommended in active IBD but could be considered for axSpA if IBD is inactive, following gastroenterology review. Dosing of secukinumab (higher dose) and ixekizumab (loading dose) differs in psoriasis. Higher (300 mg) dose of secukinumab is available for ankylosing spondylitis but not non-radiographic axSpA.

g Bimekizumab is superior to secukinumab for cutaneous psoriasis but increases incidence of candidiasis. Bimekizumab dosing differs in psoriasis (higher dose). In *post hoc* analyse of pooled axSpA trial data published after the literature search cut-off date, the bimekizumab arm had lower incidence of uveitis compared with placebo, but it is not currently licenced for uveitis. Evidence for the safety of bimekizumab in IBD is lacking.

h Tofacitinib is not licenced for non-radiographic axSpA. Tofacitinib has phase III evidence of efficacy for psoriasis but is not licenced. JAK inhibitors as a group are likely superior to placebo for uveitis according to network meta-analysis of RCTs. Tofacitinib was not superior to placebo in a phase II trial of Crohn’s.

i Upadacitinib has some evidence of efficacy for psoriasis (in PsA trials) but is not currently licenced. JAK inhibitors as a group are likely superior to placebo for uveitis according to network meta-analysis of RCTs.

**Table 2.** Components and thresholds for the Axial Spondyloarthritis Disease Activity Score (ASDAS)

Derivation	ASDAS	0.12 × back pain + 0.06 × duration of morning stiffness + 0.07 × peripheral pain/swelling + 0.11 × patient global + 0.58 × Ln(CRP + 1)
	ASDAS-ESR	0.08 × back pain + 0.07 × duration of morning stiffness + 0.09 × peripheral pain/swelling + 0.11 × patient global + 0.29 × √(ESR)
ASDAS thresholds	High disease activity	≥2.1; >3.5 indicates very high disease activity
	Low disease activity	<2.1, ≥1.3
	Inactive disease	<1.3
	Clinically important improvement	Change of ≥1.1
	Major improvement	Change of ≥2.0

ASDAS based on CRP is preferred. CRP in mg/l and ESR in mm/h. BASDAI questions and patient global are assessed on a numerical rating scale of 0–10. Patient global: ‘How active was your spondylitis on average during the last week?’.

TNF inhibitor dose reduction was not inferior to continuing standard dose for maintaining response. People with axSpA in sustained remission should be offered therapeutic tapering, with the decision agreed upon between the person with axSpA and the clinician. “Sustained remission” lacks formal definition but can be operationalized as low disease activity or remission for at least 6 months.

Flare rates were significantly higher in withdrawal arms compared with continuing standard dose or tapering arms [18]. Not all individuals who flared were able to regain control within the trial period. Although complete withdrawal is not recommended, people with axSpA, informed with these trial data, may nevertheless choose to discontinue therapy and should be supported with access to timely clinical review when needed.

### Scope questions without recommendations

The GWG did not find enough evidence with which to make recommendations on (1) the comparative safety of targeted therapies on comorbidities, and (2) the clinical effectiveness and safety of combining targeted therapies, which should be the focus of future research.

## Applicability and utility

This guideline aims to support clinical decision-making to improve quality of care for axSpA. The recommendations are intended to be pragmatic and grounded in the best available evidence but not limited by the absence of RCTs. Some recommendations diverge from existing NICE guidelines and drug licencing, which could pose barriers to implementation.

NICE recommendation to use BASDAI and spinal pain are based on historical data which are superseded by subsequent evidence for ASDAS. The overarching aim of treatment includes prevention of structural damage, for which BASDAI has lower predictive value. Implementation of ASDAS should be analogous to DAS28 thus familiar to care providers.

Questioning a diagnosis can be both practically and emotionally challenging. However, misdiagnosis is possible, particularly with the drive for earlier diagnosis. Diagnostic uncertainty, particularly in unexpected clinical trajectories, should be openly discussed within MDTs and with the person with axSpA.

Several recommendations on EMMs emphasize collaborative management with other specialties, which may not always be feasible. This emphasis hopes to provide support for combined services where there is clinical need.

Increasing the dosing interval when implementing drug tapering (except for certolizumab pegol) is outside of licencing authorization, with implications for the prescriber. However, evidence-based, shared decision-making may be more beneficial for people with axSpA than when they independently adjust dosing intervals without guidance.

The deliberate choice to prioritize non-pharmacological management at the start of the guideline for targeted therapies reflects the advocacy of individuals with lived experience and NASS. Non-pharmacological interventions are often the first and, in many cases, the only treatment required. At a time when these resources are strained or diminished, it is critical to highlight their importance in axSpA care. The GWG hopes this emphasis will support business cases for these provisions where clinical need exists.

## Research recommendations

The GWG members proposed research recommendations then voted to select the top 10 listed below.

1. Non-pharmacological management options.
2. Comparisons of targeted therapies in head-to-head clinical trials.
3. Strategies for managing fatigue.
4. Evidence on the sequential use of targeted therapies.
5. Criteria for initiating and predictors of successful therapeutic tapering.
6. Management of difficult-to-treat axSpA.
7. Role of imaging in assessing treatment response.
8. Effective use of patient-initiated follow-up.
9. Comparative safety of targeted therapies in axSpA populations.
10. Safety and efficacy of combining targeted therapies in axSpA with EMMs.

## Audit

A suggested audit tool is available via the BSR website and in Supplementary Data S6, available at *Rheumatology* online. The GWG encourages engagement with the BSR New Early Inflammatory Arthritis Audit.

## Supplementary Material

keaf090_Supplementary_Data

## Data Availability

All data are provided in online Supplementary Materials, available at *Rheumatology* online.

## References

[1] Sieper J , Poddubnyy D. Axial spondyloarthritis. Lancet 2017;390:73–84. 28110981 10.1016/S0140-6736(16)31591-4

[2] Hamilton L , Barkham N, Bhalla A et al, BSR and BHPR Standards, Guidelines and Audit Working Group. BSR and BHPR guideline for the treatment of axial spondyloarthritis (including ankylosing spondylitis) with biologics. Rheumatology (Oxford) 2017;56:313–6. 27558584 10.1093/rheumatology/kew223

[3] Zhao SS , Harrison SR, Chan A et al Treatment of axial spondyloarthritis with biologic and targeted synthetic DMARDs: British Society for Rheumatology guideline scope. Rheumatol Adv Pract 2023;7:rkad039. 37197377 10.1093/rap/rkad039 PMC10183299

[4] Tucker L , Allen A, Chandler D et al The 2022 British Society for Rheumatology guideline for the treatment of psoriatic arthritis with biologic and targeted synthetic DMARDs. Rheumatology (Oxford) 2022;61:e255–66. 35640657 10.1093/rheumatology/keac295

[5] Holroyd CR , Seth R, Bukhari M et al The British Society for Rheumatology biologic DMARD safety guidelines in inflammatory arthritis. Rheumatology 2019;58:e3–42. 30137552 10.1093/rheumatology/key208

[6] Russell MD , Dey M, Flint J et al British Society for Rheumatology guideline on prescribing drugs in pregnancy and breastfeeding: immunomodulatory anti-rheumatic drugs and corticosteroids. Rheumatology (Oxford) 2023;62:e48–e88. 36318966 10.1093/rheumatology/keac551 PMC10070073

[7] Ramiro S , van der Heijde D, van Tubergen A et al Higher disease activity leads to more structural damage in the spine in ankylosing spondylitis: 12-year longitudinal data from the OASIS cohort. Ann Rheum Dis 2014;73:1455–61. 24812292 10.1136/annrheumdis-2014-205178

[8] Poddubnyy D , Protopopov M, Haibel H et al High disease activity according to the Ankylosing Spondylitis Disease Activity Score is associated with accelerated radiographic spinal progression in patients with early axial spondyloarthritis: results from the GErman SPondyloarthritis Inception Cohort. Ann Rheum Dis 2016;75:2114–8. 27125522 10.1136/annrheumdis-2016-209209

[9] Fagerli KM , Lie E, van der Heijde D et al Selecting patients with ankylosing spondylitis for TNF inhibitor therapy: comparison of ASDAS and BASDAI eligibility criteria. Rheumatology (Oxford) 2012;51:1479–83. 22499062 10.1093/rheumatology/kes057

[10] Marona J , Sepriano A, Rodrigues-Manica S et al Eligibility criteria for biologic disease-modifying antirheumatic drugs in axial spondyloarthritis: going beyond BASDAI. RMD Open 2020;6:e001145. 32144137 10.1136/rmdopen-2019-001145 PMC7061099

[11] Overview | Spondyloarthritis in over 16s: diagnosis and management | Guidance | NICE. 2017. https://www.nice.org.uk/guidance/ng65 (26 January 2023, date last accessed).

[12] van der Heijde D , Lie E, Kvien TK et al, Assessment of SpondyloArthritis international Society (ASAS). ASDAS, a highly discriminatory ASAS-endorsed disease activity score in patients with ankylosing spondylitis. Ann Rheum Dis 2009;68:1811–8. 19060001 10.1136/ard.2008.100826

[13] Machado P , Landewé R, Lie E et al, Assessment of SpondyloArthritis international Society. Ankylosing Spondylitis Disease Activity Score (ASDAS): defining cut-off values for disease activity states and improvement scores. Ann Rheum Dis 2011;70:47–53. 21068095 10.1136/ard.2010.138594

[14] Dick AD , Tugal-Tutkun I, Foster S et al Secukinumab in the treatment of noninfectious uveitis: results of three randomized, controlled clinical trials. Ophthalmology 2013;120:777–87. 23290985 10.1016/j.ophtha.2012.09.040

[15] Sbidian E , Chaimani A, Guelimi R et al Systemic pharmacological treatments for chronic plaque psoriasis: a network meta-analysis. Cochrane Database Syst Rev 2023;7:CD011535. 37436070 10.1002/14651858.CD011535.pub6 PMC10337265

[16] Hueber W , Sands BE, Lewitzky S et al, Secukinumab in Crohn's Disease Study Group. Secukinumab, a human anti-IL-17A monoclonal antibody, for moderate to severe Crohn’s disease: unexpected results of a randomised, double-blind placebo-controlled trial. Gut 2012;61:1693–700. 22595313 10.1136/gutjnl-2011-301668 PMC4902107

[17] Molto A , López-Medina C, Van den Bosch FE et al Efficacy of a tight-control and treat-to-target strategy in axial spondyloarthritis: results of the open-label, pragmatic, cluster-randomised TICOSPA trial. Ann Rheum Dis 2021;80:1436–44. 33958325 10.1136/annrheumdis-2020-219585 PMC8522451

[18] Landewé RB , van der Heijde D, Dougados M et al Maintenance of clinical remission in early axial spondyloarthritis following certolizumab pegol dose reduction. Ann Rheum Dis 2020;79:920–8. 32381562 10.1136/annrheumdis-2019-216839 PMC7307216

